# Integration of High-Volume Molecular and Imaging Data for Composite Biomarker Discovery in the Study of Melanoma

**DOI:** 10.1155/2014/145243

**Published:** 2014-01-16

**Authors:** Konstantinos Moutselos, Ilias Maglogiannis, Aristotelis Chatziioannou

**Affiliations:** ^1^Department of Computer Science and Biomedical Informatics, University of Thessaly, Papasiopoulou 2-4, 35100 Lamia, Greece; ^2^Department of Digital Systems, University of Piraeus, Grigoriou Lampraki 126, 18532 Piraeus, Greece; ^3^Metabolic Engineering and Bioinformatics Programme, Institute of Biology, Medicinal Chemistry and Biotechnology, National Hellenic Research Foundation, 48 Vasileos Constantinou Avenue, 11635 Athens, Greece

## Abstract

In this work the effects of simple imputations are studied, regarding the integration of multimodal data originating from different patients. Two separate datasets of cutaneous melanoma are used, an image analysis (dermoscopy) dataset together with a transcriptomic one, specifically DNA microarrays. Each modality is related to a different set of patients, and four imputation methods are employed to the formation of a unified, integrative dataset. The application of backward selection together with ensemble classifiers (random forests), followed by principal components analysis and linear discriminant analysis, illustrates the implication of the imputations on feature selection and dimensionality reduction methods. The results suggest that the expansion of the feature space through the data integration, achieved by the exploitation of imputation schemes in general, aids the classification task, imparting stability as regards the derivation of putative classifiers. In particular, although the biased imputation methods increase significantly the predictive performance and the class discrimination of the datasets, they still contribute to the study of prominent features and their relations. The fusion of separate datasets, which provide a multimodal description of the same pathology, represents an innovative, promising avenue, enhancing robust composite biomarker derivation and promoting the interpretation of the biomedical problem studied.

## 1. Introduction

Integration of multimodal and multiscale data is of known importance in the context of personalized medicine and future electronic health record management. The quest for suitable data fusion schemes, which could ideally optimize the exploitation of the information residing in composite datasets, is an emergent area with numerous potential applications. In the context of Virtual Physiological Human (VPH), an integrated framework should promote the interconnection of predictive models pervading different scales, with different methods, characterized by different granularity. Such a framework consolidates system level information and enables formulation and testing of hypotheses, facilitating a holistic approach [1].

In this work we propose a novel methodology on multimodal data fusion regarding separate datasets. As separate we define datasets where each has been obtained from a different technological source and from a different set of patients. Even the number of patients participating in each examination is not the same. The only common determinant of separate datasets is that they refer to the same disease. Such datasets are not amenable to ordinary fusion methods, as all the known methods deal with the same set of patients being examined by various instruments and techniques in sequence, thus producing the multimodal data. However, the majority of open-accessible data refers to the unimodal results of certain experiment relative to a specific disease. The suggested methodology is able to highlight biomarkers utilizing these separate unimodal outcomes and thus repurpose the existing data of accessible repositories.

As proof of concept, we focus on the fusion of two separate unimodal datasets, one of molecular and one of imaging description, both concerned with the study of cutaneous melanoma (CM). Application of feature selection and dimensionality reduction algorithms on the produced unified dataset can contribute towards the extraction of better biomarkers, ruling out false positive findings coexisting, but with no causal association, with the investigated disease. The procedure can be applied to various cases and tackle separated datasets of other diseases as well.

This paper is organized as follows: Section 2 includes related work and background on information fusion methods, the cutaneous melanoma disease, and the feature selection techniques used in this work.  Section 3 contains the preprocessing steps for the preparation of the unimodal datasets, the construction of the unified table with the use of imputation methods, and the implementation details of the feature selection methods regarding random forest, principal component analysis, and linear discriminant analysis.  Section 4 encloses the results of the feature selection procedures regarding specific biomarkers and their performance and stability observed during repetitive runs. Finally, in Section 5 we discuss the use of synthetic data via the simple class imputation methods, the multiple imbalances present at the joined datasets, a comparison considering the modal origin of the highlighted features, and biological implication of the proposed biomarker sets. The paper concludes with future work.

## 2. Background and Related Work

### 2.1. Information Fusion

Information fusing algorithms can be classified as belonging to one of the following categories: Combination of Data (COD) or Combination of Interpretations (COI) [2]; COD methods aggregate features from each source into a single feature vector before classification, while COI methods classify the data from each source independently and then aggregate the results. Rohlfing et al. [2] compared the two methods to combine information sources in different biomedical image analysis applications, while Haapanen and Tuominen [3] followed a COD approach for the combination of satellite image and aerial photograph features for higher accuracies at forest variable estimation. On the other hand, Jesneck et al. [4], on a COI path, optimized clinically significant performance measures in a decision-fusion technique combining heterogeneous breast cancer data. Lee et al. [5] proposed a Generalized Fusion Framework (GFF) for homogenous data representation and subsequent fusion in the metaspace, using dimensionality reduction techniques. The metaspace comprises the projections of the heterogeneous data streams transformed in a way that scale and alleviate dimensionality differences. Such metaspace representation approaches, which transform data into a homogeneous space enabling direct combination of modalities, are embedding projections and kernel space projections [6]. For example, Hinrichs et al. [7] used multikernel learning, while Gray et al. [8] used manifolds and random forests to exploit multimodal datasets on Alzheimer's disease. The first work resulted in a multimodal disease marker to predict the progress of the disease, while the second resulted in a model, which outperforms the predictions of the individual unimodal datasets. Golugula et al. [9] applied a specialised version of regularized canonical correlation analysis on histologic imaging and proteomic signatures regarding prostate cancer data, for the creation of a metaspace representation. Utilizing random forests they were able to predict biochemical recurrence of the disease with significantly higher accuracy.

GFF algorithms assume that we have raw data from sources *S*_*i*_(*x*_1_, *x*_2_,…, *x*_*k*_), where *x*_1_, *x*_2_,…, *x*_*k*_ represent the *k* observations in a study (i.e., rows in a dataset) and *i* represents one of the *N* data sources, *i* ∈ {1,2,…, *N*}. While this could be the case for specific studies or electronic patient records, most available datasets are essentially unimodal. That is, there are single modal data for a set of patients and then other available data of a different modality from observations on a different set of patients; both sets refer to the same disease although possibly at different stage or phase of the disease. Hence, there are other sources *S*_*j*_(*y*_1_, *y*_2_,…, *y*_*l*_), *j* ∈ {1,2,…, *M*}, where in general *l* ≠ *k* and *M* ≠ *N*. Nonetheless, the description conferred by each technological source *S*_*i*_, *S*_*j*_ characterizes the same disease, implying common phenotypic manifestations. Furthermore, associations underlining causal biological actions could emerge due to associated physiological determinants. We name the datasets derived from the sources *S*_*i*_ and *S*_*j*_ as *separate* datasets.

### 2.2. Cutaneous Melanoma

Cutaneous melanoma (CM) is considered a complex multigenic and multifactorial disease that involves both environmental and genetic factors. It is the most life-threatening neoplasm of the skin, and its incidence and mortality are constantly increasing worldwide. CM tumorigenesis is often explained as a progressive transformation of normal melanocytes to nevi that subsequently develop into primary cutaneous melanomas (PCM). However, the molecular pathways involved have not been clearly elucidated, although considerable progress has been made [10]. Despite the success of genomics in defining genomic markers or gene signatures for other kinds of cancers (such as breast cancer), there has been no similar progress related to malignant melanoma.

The microarray studies that have been performed on CM by different groups exploit different microarray technological platforms applied in highly heterogeneous patient cohorts and pathological sample collections [11]. These differences hurdle significantly comparisons, yielding cohorts of reduced total size and diversity. Integration of independent cohorts from different studies bears significant challenges for a number of reasons stemming from the technical design to purely biological ones [12].

Regarding the clinical methods for diagnosis of melanoma, there exist several standard approaches for analysis and diagnosis of lesions, For example, the Menzies, scale, the Seven-point scale, the Total Dermoscopy Score based on the ABCD rule, and the ABCDE rule (Asymmetry, Border, Color, Diameter, Evolution). In these methods, digital images can serve as a basis for the medical analysis and diagnosis of lesions under consideration. As human interpretation of image content is fraught with contextual ambiguities, advanced computerized techniques can assist doctors in the diagnostic process [13]. A review of image acquisition and feature extraction methods utilized in the literature regarding existing classification systems can be found in [14].

### 2.3. Feature Selection

Feature selection techniques do not alter the original representation of the input variables but merely select a subset of them, a contrast to other dimensionality reduction techniques like those based on projection (e.g., principal components analysis) or compression (e.g., using information theory). Thus, feature selection techniques preserve the original semantics of the variables, enhancing interpretability by a domain expert [15].

The main objectives of feature selection are (a) to avoid overfitting and improve model performance, (b) to provide faster and more cost-effective models, and (c) to gain a deeper insight into the underlying processes that generated the data.

Regarding the applied feature selection procedures in this study, at first, a wrapper type technique was employed (sequential backward elimination—SBE) using the random forest (RF) algorithm [16], which utilizes ensembles of decision trees. SBE algorithm starts with the full set of features and iteratively removes the feature computed as least important each time, until a required number of features remain. As an option, a multivariate filter was used to reduce the colinearity among features of the microarray dataset, prior to the application of the wrapper method. This filtering together with the imputation represents a transition from a COD method towards a GFF approach, although here no further transformation is applied to the feature vectors.

The random forest algorithm, among other ensemble learning methods, is reported to be successful in variance reduction, which is associated with reducing overfitting [17]. In addition, we used the option of stratifying the bootstrapped samples with equal number of cases per class [18]. This is compatible with the Balanced Random Forest (BRF) approach, which is computationally more efficient with large imbalanced data, since each tree only uses a small portion of the training set to grow. Additionally it is less vulnerable to noise (mislabelled class) than the Weighted Random Forest (WRF) where a heavier penalty is placed on misclassifications of the minority class [19]. BRF alleviated the class imbalance problem, a common problem in disease diagnosis, where the disease prevalence entails that disease cases are a small fraction of the total population. In our case dermoscopy and microarray data had class imbalance ratios of disease to healthy samples: 70 : 972 and 45 : 18, respectively. The recognition goal is to detect people with the disease; thus a favorable classification model is one that supports higher identification rates for the disease category. Additionally, the random forest algorithm was chosen for this study for one more reason: the method has only two optimizing parameters, the number of created trees as an ensemble and the number of randomly tried features on each split as a tree is created.

Next, principal component analysis (PCA) and linear discriminant analysis (LDA) were employed to highlight top features with larger coefficients as potential biomarkers. PCA is an unsupervised method and a data reduction technique that allows the major sources of variation in a multidimensional dataset to be analyzed without introducing inherent bias. PCA as a manifold provides an isomorphic, direct mapping of high-dimensional data into a lower-dimensional space capturing and representing the information of the original data incrementally. PCA defines new orthonormal in-between variables, consisting of linear combinations of the original variables such that the axis of the first principal component (PC) highlights the dimension collinear to the most variation and the axis of the second component the dimension with the most of the remaining variation and so on. The coordinates of the samples in the new space created by the PCs are called scores [20].

LDA uses class information to maximize the separation between various groups of observations. LDA presumes that the classification variables follow a normal multivariate distribution and the covariance matrices for the observations of each class are equal (homoscedasticity). When these working assumptions are not considered plausible, LDA does not represent the optimal classifier. However, it can still be considered a valid and accurate method for the screen of the multidimensional solution space, when the objective is the determination of separating hyperplanes that maximize discrimination between different classes. This is so, because the hypotheses on the form of the data distributions do not have any impact on the solution of the geometrical separation problem [21].

## 3. Materials and Methods

### 3.1. Multimodal Data Fusion of Separate Datasets

**Figure 1:**
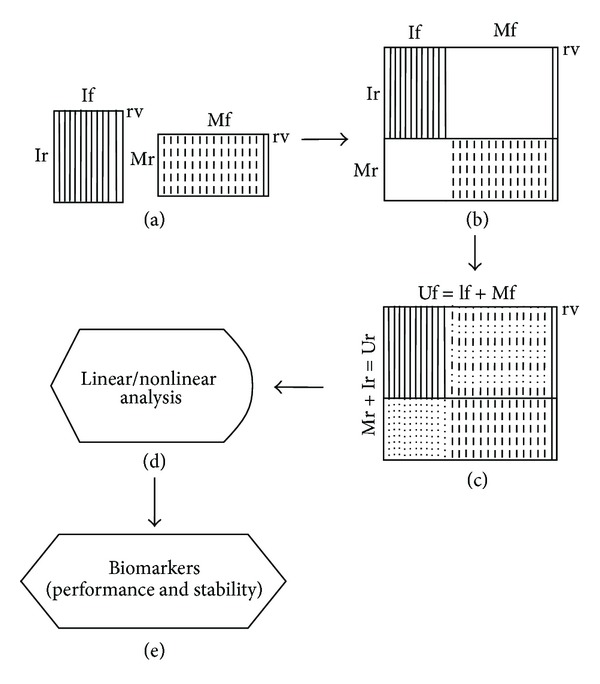
Data fusion workflow for separate datasets: (a) separate datasets, (b) unified sparse dataset, (c) unified dataset (class imputations), and (d) and (e) multivariate statistical analysis and feature selection. See text for details.

The workflow of the methodology is shown in Figure 1. At the initial phase (a) there are two unimodal separate datasets (image and microarray data). Each table has different features (If and Mf) and different numbers of observations/rows (Ir and Mr) obtained from different patients. The last column of the tables represents the response variable (rv). In our case it is a binary response variable with two classes: healthy or disease. Next (b) the unimodal tables are merged to one block sparse matrix. The only column without nonavailable values is the one with the response variable. Subsequently, at step (c) simple biased imputations are performed per feature and per class. These are depicted with dotted lines. The total number of rows and features of the unified table is the sum of the rows and features of the two initial tables (Ur, Uf). Now the table is amenable to multivariate statistical analysis, and specific composite biomarkers can be extracted and studied for their performance contribution and stability in their appearance over repetitive runs of synthetic data creation via the imputations.

All workflow programming was implemented in R [22].

#### 3.1.1. Image Data

The dataset derived from skin lesion images contained 972 instances of nevus skin lesions and 69 melanoma cases. Three types of features were analyzed: Border Features which cover the A and B parts of the ABCD-rule of dermatology, Color Features which correspond to the C rules, and Textural Features which are based on D rules. 31 out of the initial set of 32 possible features were used; one feature was removed due to having zero variation across the samples. The relevant preprocessing for all features is described in [23]. The dimensions of the image dataset were thus 1041 (rows) × 31 (columns).

#### 3.1.2. Microarray Data

The microarray dataset was taken from the Gene Expression Omnibus (GEO) [24], GDS1375. In that experiment, total RNA isolated from 45 primary melanoma, 18 benign skin nevi, and 7 normal skin tissue specimens was used for gene expression analysis, using the Affymetrix Hu133A microarray chip containing 22,000 probe sets [25]. The dataset contains the MAS5-normalized signal intensities and is globally scaled so that the average intensity equals 600.

Data retrieval from GEO was performed using GEOquery [26] and concomitantly processed with limma [27] R packages from the Bioconductor project [28], following the proposed steps as listed in the R script produced by the GEO2R tool [29]. The gene expression values across all categories were log-transformed, and the mean values of all genes in the normal skin were calculated. Subsequently, the mean gene vector concerning the normal skin categories was subtracted from all replicate vectors of the other two categories. In this way, the initial signal intensities provided ratios of differential expression, calculated by dividing the signal intensities of each category by the respective gene value of the normal category. As all values have been log-transformed, the division is replaced with a subtraction. For the remaining analysis the differentially expressed gene values of the melanoma versus skin and nevi versus skin were exploited. 1701 genes from a linear model fit were extracted setting FDR for multiple testing adjustment, *P* value 0.001 and 2-fold changes as thresholds. The dimensions of the microarray dataset were thus 63 (rows) × 1701 (columns).

#### 3.1.3. Data Integration

The two tables containing the microarray and image data were merged to one block sparse matrix with dimensions 1104 rows × 1734 columns, marking the unavailable values as NA. The rows contain the microarray and image data samples and the columns microarray and image features plus one binary response variable (0 for nevus and 1 for melanoma).

#### 3.1.4. Missing Values Imputation

Although there are several software packages implementing advanced imputation methods [30], they could not be utilized in this unified dataset where the multimodal data have only the class variable column as complete. In this study we considered four simple imputation methods applied per feature and per class:

- “mean value” imputation,
- “random normal” imputation,
- “uniform” imputation,
- “bootstrap” imputation.

In the second case, after estimating the mean value (*m*) and standard deviation (sd) of each feature (ignoring the NA values) per class, we randomly filled the missing values sampling from an assumed normal distribution having as parameters: (*m*, sd). The “uniform” imputation is conducted by sampling uniformly within the range of each feature per class and the “bootstrap” imputation by independent bootstrap of each variable separately per class, until all the NA values are replaced. The last two imputation methods are similar to the way random forests construct synthetic data, in order to provide for a similarity measure [18]. For the efficient execution of the imputations, the plyr R package was employed [31].

### 3.2. Feature Selection with Random Forest

The first feature selection workflow was built, using the R package caret (classification and regression training) [32]. The search algorithm employed in caret uses the recursive feature elimination method on predefined sets of predictors and in this study the length of the variable subsets was defined as [1 to 10, 15, 20, 25, 30, 35, 40, 45, 50], except for the image-only data, where the subsets were [1 to 10, 15, 20, 25, 30, 31] due to the number of image predictors.

The setup of the insilico simulation involved the examination of the reported selected feature subsets when (a) applying a colinearity removal filter to the microarray dataset prior to the execution of the selection algorithm (marked as filtered/unfiltered), and (b) setting a 95% tolerance threshold to the best-obtained performance criterion (Tolerance/Best). As noted at the caret documentation, the colinearity filter computes the correlations between the microarray features and then screens the correlation matrix, in order to remove features with high pairwise correlation. If two variables have a high correlation (0.75 is set by default as cut-off value), the function traces the mean absolute correlation of each variable and removes the variable with the largest mean absolute correlation. The default cut-off value was empirically selected as to reduce the number of high-correlated features (genes) up to a level where “enough” genes were left to continue with the analysis (~30%). The cut-off value is dataset dependent, as the correlation among the features of an experiment is inherent to the experiment itself. As to the tolerance-in-the-performance criterion, it allows the selection of a subset size that is small enough but without sacrificing too much performance and can yield good results with a performance plateau for larger subset sizes. The combination of prior-filtering and tolerance-threshold resulted in the examination of four distinct cases: “Filtered Tolerance,” “Filtered Best,” “Unfiltered Tolerance,” and “Unfiltered Best.”

For each of the four cases, a 10-fold cross-validation procedure was performed with 50 repetitions on six different datasets:

- only the microarray data (marked as o.m),
- the unified dataset produced by the mean imputations (m.i),
- the unified dataset by normal random imputations for the NA values (nr.i),
- the unified dataset by the “uniform” imputations (u.i),
- the unified dataset by the “bootstrap” imputations (b.i),
- only the image data (o.i).

Throughout all repetitions, the nr.i, u.i, and b.i datasets were reimputed, thus providing more sampling variations. Prior to the application of the repetitions, the datasets were centred and scaled.

For each of the 50 repetitions, the cardinality of the optimum subset of predictors was recorded, along with the names of the predictors and the performance attained. The area under the ROC curve (auc) was used as a performance metric. The auc of a classifier is equivalent to the probability that the classifier will rank a randomly chosen positive instance higher than a randomly chosen negative instance. This is equivalent to the Wilcoxon test of ranks and is also closely related to the Gini coefficient [33].

### 3.3. Feature Selection Using Linear Multivariate Statistical Analysis

Statistical analysis was performed using multivariate techniques, specifically PCA, followed by LDA. For all the cases, the colinearity removal filter from the caret package was applied to the microarray data. PCA was performed by the prcomp R function and LDA by the lda function from the MASS R package [21].

The lda function has two working modes: one having the parameter CV = False (the default), implying no application of cross-validation methods, meaning it can obtain an object that includes discriminant scores, and the other with CV = True, where predictions of class memberships are derived from leave-one-out (LOO) cross-validation. In LOO CV a model is trained iteratively with all the available observations (rows) but one each time, and a prediction is made as regards the response value of the left out observation. This procedure provides an estimation of the overall performance of our model.

Initially, we ran the lda function on each of the examined datasets and retrieved the estimated scores as well as the Singular Value Decomposition (SVD) parameters. SVD parameters provide the ratio of the in-between- and within-group standard deviations on the linear discriminant variables. Their squares conform to the canonical *F*-statistics. The lda function (having CV = True) was executed 50 times on each dataset (value imputations were applied each time to the unified datasets), in order to assess the variability of the attained accuracy performance for the melanoma class. Next, the lda function was run again 100 times (having CV = False) on each dataset in order to assess the stability of the suggested top performing features.

Finally, the set of the 20 top scoring biomarkers was derived, in order to assess the prediction performance for the melanoma case. The LDA (CV = True) and RF (stratified, 18 samples per class) methods were applied 50 times on each dataset using only the biomarkers' columns and the class as response variable. The RF performance was assessed using the out-of-bag (OOB) error estimation. OOB are the rows of the dataset that have not taken part in the creation of a decision tree due to the bootstrap procedure, and therefore they can be used to assess the performance of the created decision tree.

## 4. Experiments and Results

### 4.1. Feature Selection with RF

**Figure 2:**
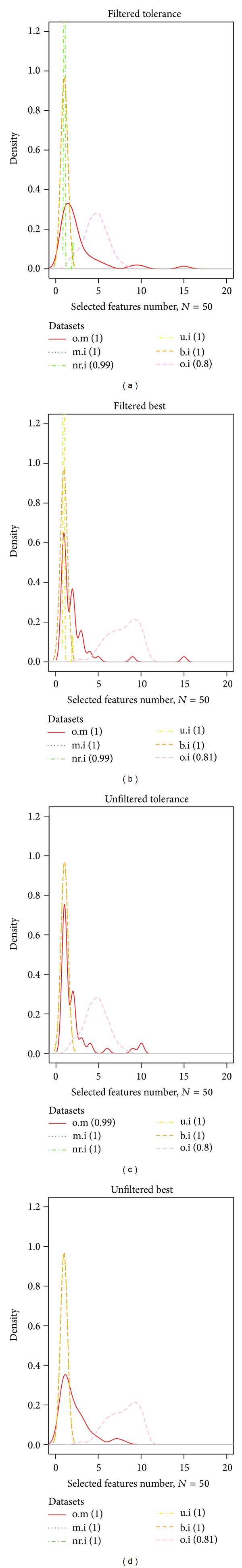
Gaussian kernel density plots of the optimum features number from 50 repetitions. The six datasets are only microarray (o.m), mean imputation (m.i), normal random imputation (nr.i), uniform imputation (u.i), bootstrap imputation (b.i), and only image (o.i). In parentheses are the medians of the obtained performances (auc) for each dataset.

The results of the tests regarding the feature selection process are depicted in Figure 2. All missing value imputation schemes applied onto the unified datasets yielded an almost perfect score as can be surmised from the median values of the area under curve (auc) estimates of those datasets. These median auc values are displayed in parentheses for each dataset at the data-label boxes in Figure 2.

The unimodal dataset of the image data (o.i) exhibited lower performance scores together with the higher cardinality of the selected features set. The application of the colinearity reduction filter to the microarray data had little effect regarding the dispersion of the optimum subset cardinality. The execution time in the condensed dataset, derived after the application of the filter, however, was 4 times faster than before, in proportion with the reduced number of remaining features after the use of the filter (482 from the initial 1701 differentially expressed genes in the microarray dataset). The results on the imputed datasets of filtering demonstrated a drastic reduction regarding the number of features required to build the classifier, as well as the constancy of the feature subsets, regarding the derivation of the classifier in the iterative framework.

The four imputed datasets, despite their different mechanism of imputed value generation, produced classifiers with similar cardinality. As shown in the feature of Tables 1 and 2, the normal random imputation dataset (nr.i) resulted in a considerably more stable selection of features compared to the mean imputation unified dataset (m.i). The same pattern is observed for the unfiltered cases (data not shown). In the unfiltered cases, the nr.i dataset exhibited far better stability in the formation of the predictor set, outperforming even the unimodal transcriptomic (microarray-only) dataset. This improvement supports the expediency of coming up with an extended integrative dataset as this approach stabilizes the performance and tackles covariance effectively, managing to rescue the critical information that enables correct classification.

The features from the mean-imputation unified dataset presented higher instability than all other methods and so proved to be the least preferable approach for the imputation procedure. All other imputation schemes (nr.i, u.i, and b.i) performed equally well in terms of stability.

The noted weaknesses in the use of solely performance indicators for marker discovery, without considering the stability of the proposed marker set, has been raised in the literature [34] and is in congruence with the findings of this study. The imputations applied using the nr.i, u.i, and b.i schemes resulted in a balanced selection of the predictor set from the derived, unified dataset each time. This was attained through the expansion of the feature set and thus the neater representation with respect to its stratification of the total information variation of the experiment. Consequently, this resulted in the retrieval of smaller optimum subsets of features, encompassing at the same time a more stable selection of genes to be considered as candidate biomarkers.

#### 4.1.1. Image-Derived Features Importance by RF

**Figure 3:**
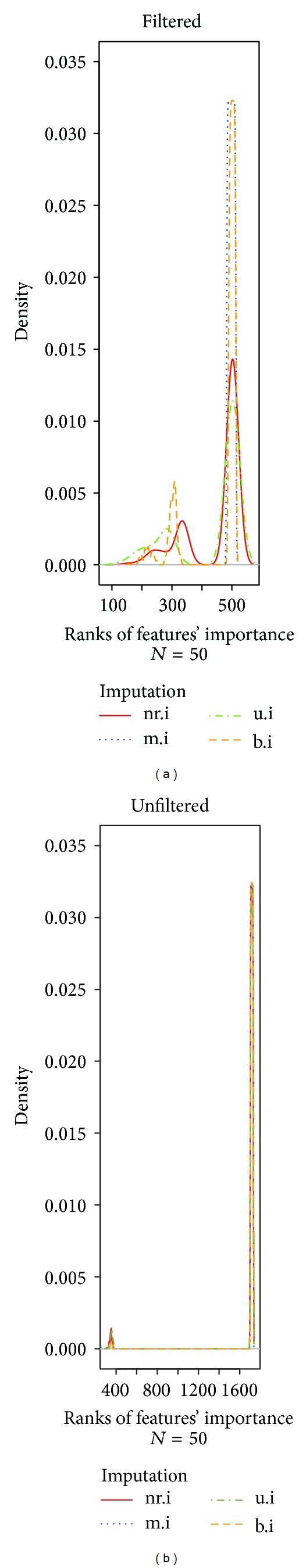
Kernel density plots of importance ranks for image-derived features on the unified dataset. Ranking in the *x*-axis is in decreasing order of importance.

Notably, none of the image-derived features were present in the top selected features of the unified datasets, as shown in Tables 1 and 2. In order to assess the consistency of the ranking of image features, 50 repetitions of the random forest algorithm were executed for each of the unified datasets derived by the four imputation methods (m.i, nr.i, u.i, and b.i). Each of the resulting 50 lists of features was sorted by decreasing importance. Next, the positions of the image features in the lists were collected; the density plots for the filtered/unfiltered cases are shown in Figure 3. Random forest exposes four importance measures [35] and in this case the “Mean Decrease Gini” criterion was chosen. The results using the other three criteria were similar (data not shown).

The majority of the image features were ranked as less important when compared to the features from the microarrays; this implies their lower informative power concerning the total observed variation in the integrated dataset. The lower informative power of the image features could be attributed to the phenotypic complexity of the image feature space, as well as the technical covariance and the size of its feature space. These factors mark their fingerprint in the integration process, despite the application of normalization techniques, thus impacting the response vector of the disease. When using the nr.i, u.i, or b.i method, however, the image features perform better, demonstrated through their more frequent presence in higher positions of the classifier's vector. This contrasts with the results of the m.i method. The mean imputation process relegated all image features to the lowest positions of the complete feature set, considering them less informative compared to the microarray features. In this sense, it is obvious that the three imputation methods, nr.i, u.i, and b.i, yield a more impartial effect. This can be surmised from the improved score of the image related features, providing practical value to its application in the integration process. The simulated dataset thus derived has a more balanced representation of features from the two modalities (microarray and image).

In order to assess whether the small size of the image features (31 variables) compared to the 482 microarray features (after the colinearity filtering from the initial 1701 microarray features) is the reason that RF algorithm consistently favors microarray predictors as the best performers, we performed a series of simulations similar to those described in Figure 3. This time, however, for the derivation of the unified datasets we replicated the image features 10 times, so adding 310 replicated image features, in order to balance the feature size effect with the microarray features. The results again showed the same preference to the microarray features, excluding the case that the behavior shown in Figure 3 was due to the feature size imbalance.

### 4.2. Feature Selection with PCA and LDA

**Figure 4:**
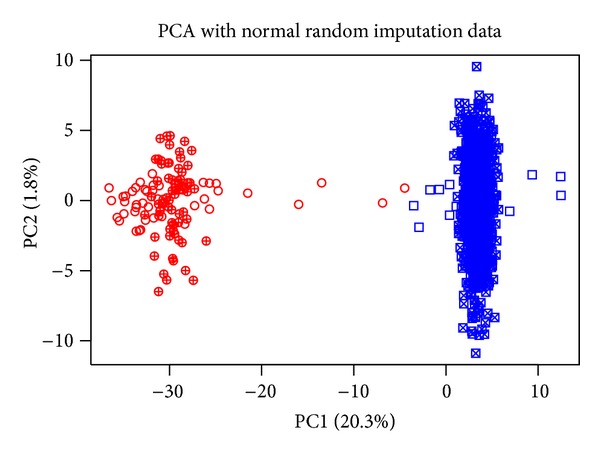
Scores plot for the nr.i unified dataset. In parentheses is the percentage of variation covered by each principal component. With circles are the melanoma samples (red), and crossed (either circles or rectangles) are the image data points.

In Figure 4 the representation of the dataset (scores plot), using the first two principal components after the nr.i imputation method, is shown. The first principal component, which describes the largest part of the data variation, can discriminate the melanoma/nonmelanoma classes quite well. Comparing the graphs of the PCA for all the cases (Supplementary 1 in Supplementary Material available online at http://dx.doi.org/10.1155/2014/145243) we note that while in unimodal image data more than 2 PCs are needed in order to discriminate the two classes, in all other cases PC1 can separate the classes, with the exception of 2-3 samples, all of which come from the microarray dataset (noncrossed symbols). The three imputation methods (nr.i, u.i, and b.i) perform similarly regarding the extent of separation they attain between classes, as well as the percentage of variation captured by the two PCs.

**Figure 5:**
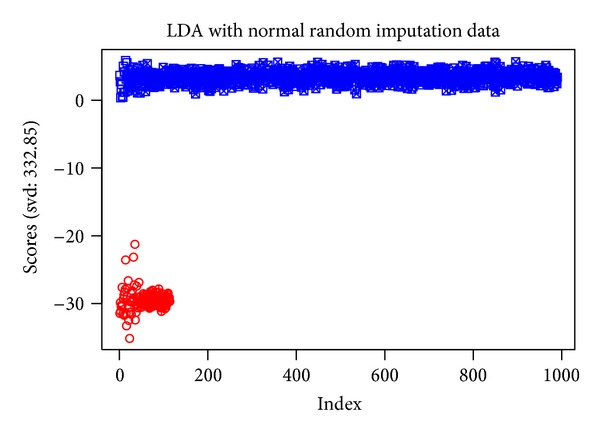
LDA scores plot for the nr.i dataset. With circles are the melanoma samples, and crossed (either circles or rectangles) are the image data points.

The plot of the scores after application of the LDA after the nr.i imputation method is shown in Figure 5. Comparing the graphs of the LDAs, this time for all the datasets (Supplementary 2), it is obvious that the imputation process significantly increases the discrimination between the two classes, as denoted by the svd values. The mean imputation process yields the smallest increase of the SVD value relative to nr.i, u.i, and b.i procedures.

**Figure 6:**
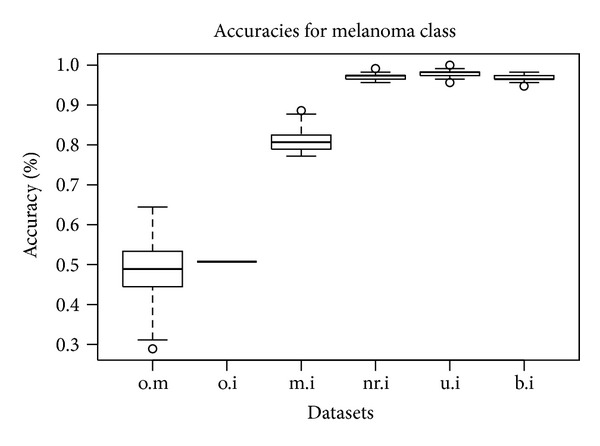
LDA CV accuracy for the melanoma class for each dataset (*N* = 50).

The execution of the lda function 50 times in LOO cross-validation mode for each of the datasets (unimodal or integrative according to the different imputation schemes) enables measurement of the accuracy of the predictive model for the case of melanoma class. The accuracy is measured as the percentage of correct guesses in all predictions made by the model related to the dataset. As it is shown in Figure 6, the accuracy for the nr.i, u.i, and b.i datasets is very high (over 95%). The mean imputation of unified dataset again underperforms compared to the other three imputation methods. There is a significant improvement in the attained LDA accuracy regarding the accuracy of the original microarray-only and image-only datasets.

As with the high performing features derived by the application of the random forest algorithm, the stability of the features suggested by the LDA is of high importance. In order to test the stability of the features derived by the LDA, we executed the lda function 100 times without cross-validation for each integrative dataset and recorded the LDA coefficients (loadings). Then we introduced two performance indicators that rank feature performance as follows: the top-20 features with the largest mean coefficient (*top-means*) or the top-20 features which appeared most of the times having the top-20 largest coefficients (*top-20*). The results are shown in Table 3.

In Table 3 the three first columns (o.m, o.i, and m.i *top-means*) are retrieved after only one run, since there is no variation in the LDA coefficients as the dataset is stable in these cases. The top*-*20 features for o.m, o.i, and m.i have been included so as to have a full view of the best variables at each dataset. As it is obvious from the frequencies columns, the three imputation methods (nr.i, u.i, and b.i) show similar distributions. In order to assess the method which presents the better stability, we used as *stability indicator* (si), the number of common features between *top-means* and *top-20 *columns for each method. The si for nr.i, u.i, and b.i was 2, 5, and 1, respectively.

**Figure 7:**
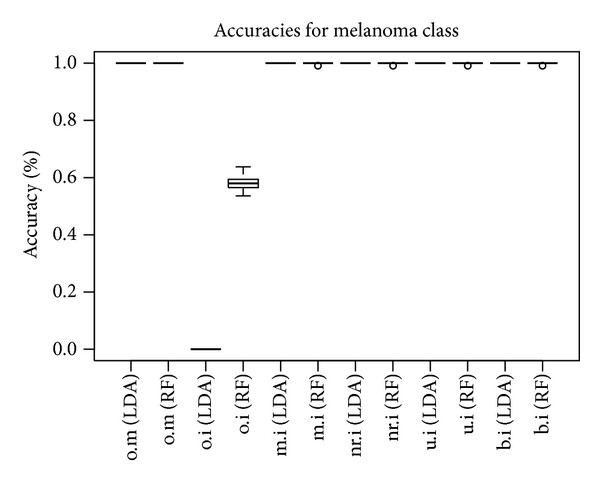
LDA LOO CV and RF OOB performance using u.i *top-means* biomarkers (*N* = 50).

As a last step, we assess the predictive performance of the top features (u.i *top-means*), looking at the LDA and RF algorithms, to all the datasets. For the cases of the original datasets (o.m, o.i) only the relative part of the biomarkers was used as predictors. The results are shown in Figure 7. A perfect score is achieved by random forests for all the datasets apart from the image-only original dataset. LDA reports an almost perfect score too, apart from the o.i dataset in which it cannot guess correctly any of the melanoma samples.

## 5. Discussion

In this work we propose the fusion of two *separate* datasets, concerning the same disease, as a new approach for the extraction of better biomarkers. The study extends the evaluation of the optimum set of predictors not only in light of the attained performances but in relation to the stability of the resulted predictors as well. This is the first attempt, to the best of our knowledge, to assess feature selection algorithms on integrative datasets retrieved from separate sources (modalities), where each source comes from a different set of patients, connected however by the same pathological mechanism. Although this study focused in the CM disease, the method could be applied, in general, to any disease where separate datasets exist, such as those residing in microarray expression, proteomic and genomic data repositories. Of course, special care has to be given to the selection of matching experiments regarding the disease, so samples, preparation methods, and the subsequent analysis are in the same context. In this work regarding CM, for instance, the microarray experiment selected was carefully screened among dozens residing in the GEO related to melanoma in order to match comparable state, progress, and tissue taken for the disease (e.g., no artificial cell lines, not only metastatic tumours, etc.).

### 5.1. Use of Synthetic Data

The application of imputation methods per feature and per class fills the gaps in the block sparse matrix of the unified dataset and produces a dataset which is amenable for processing by multivariable machine learning methods. The imputation process as performed here is class dependant and so inserts a significant bias at testing time towards achieving high performance accuracies. As a heavy biased procedure still, it is able to integrate the different datasets at the data level and highlight features and relations amongst them which would otherwise be impossible.

The three imputation methods (apart from the mean imputation), where each relies on a given statistical distribution scheme, perform similarly (auc or accuracy) regarding the predictor sets (biomarkers) they propose for the 2-class classification problem. These methods also performed equally well regarding the stability of the predictors, seen especially in the RF results. In all cases the unified datasets were able to produce biomarkers of higher predictive performance (with the exception of the RF case for the only-microarray data, which produces analogue results). In particular, when the stability of the proposed biomarkers was taken into account, the unified datasets had a superior performance, especially when compared with the solely microarray related features.

### 5.2. Dataset Idiosyncrasies

The two joined datasets are characterised by class imbalance, features imbalance, and imbalance of the number of observations between the modes. An additional caveat is that the columns in both datasets are covariant. The random forest algorithm, applied in stratified bootstrapped samples, was capable of tackling these issues and performs almost perfectly in most cases, with the exception of the image-only data, which had an auc score of 0.8. The linear approaches (PCA and LDA) did not perform highly in the cases of the unimodal datasets (image or microarrays). For the unified datasets, however, both RF and LDA attained top scores.

### 5.3. Impact of the Unimodal Datasets

One of the noticeable differences in the proposed predictor sets between RF and LDA is the performance of the image features. RF indicates that all image features score lower than the 100th position of importance for the filtered case or after the 350th position for the unfiltered case. In the linear analyses of PCA and LDA, however, presence of image features in the predictor sets is on a par with the microarray features. In addition, from the two used stability indicators on LDA, *top-means* favours microarray features (e.g., 17 to 3 for the u.i *top-means*), and top-20 favours image features (e.g., 2 to 18 for the u.i top-20). Moreover, as surmised from the svd values (Supplementary 2) of the unimodal datasets, both perform similarly and so the proposed top predictor sets contain variables from both modes. Additionally, in the same linear perspective, the image data points present better class separation in the unified datasets, as denoted by the crossed marks in the PCA and LDA scores plots. The selection of the 20 u.i *top-means* set as biomarkers (as seen in Figure 7) resulted in an almost perfect performance on all the datasets for both methods (RF and LDA) apart from the image-only case. This is due to the fact that the selected u.i *top-means* set contains only 3 image features. RF, however, needs between 5 to 10 image features to reach the top performance as seen in Figure 2 for the two “best” cases. This is even more obvious for the LDA method, which fails to predict any melanoma case from the 3 proposed image predictors, as seen in Figure 7.

These findings support the notion that the expansion of the feature set, through the use of the imputation methods, benefits the classification process. Proposing geometric spaces where appropriate separating hyperplanes can be derived incurs good performance in a methodologically similar way with methods as the Support Vector Machines. It is worth noting the dramatic improvement of the predictor sets inferred by the unified datasets (although biased), in terms of classification performance as well as informational content regarding the explained variation. Of paramount importance is the fact that this superior performance is rescued even by subsequent application of drastic data reduction techniques, such as the *top-means* or the top-20 formalisms. A possible explanation for this finding is that the transformation of the initial classification problem in a geometric space, which alleviates features covariance, makes leeway for the inference of separating planes between classes that perform robustly. This improvement is therefore retained permanently, even when the representation space is collapsing; a different, however, still robust combination of features that define the signature set is feasible. Moreover, through the integration of data from two different layers of organization of the biological information (molecular and whole tissue), the effect of noise, in the form of false positives in each layer of description, is seriously limited. By transforming the initial datasets into a unified phantom set the effect of arbitrary feature covariance due to noise is confined in this layer of organization, downgrading their impact in the unified dataset and thus disqualifying them from candidates for the predictor set.

### 5.4. Biological Implications of the Biomarker Sets

The analysis of the composite multimodal signature sets presented in Table 3, according to the two different performing indicators, *top-means* and *top-20*, proposes equivalent feature sets capable of classifying robustly and accurately human samples, probed either through molecular (gene expression profiling) or imaging (dermoscopic evaluation) examination. The fact that these signatures are top performers, not only in terms of efficiency in classification but also regarding the informational content of the observed variation that they manage to explain in the integrative dataset, renders them ideal starting points for functional interpretation of critical determinants of the CM pathophysiological mechanism. As previously said those signatures bear complementarity regarding the modules they select in order to perform the classification task, in terms of functional implication. Interestingly, the *top-means* indicator, which comprises the 20 features with the largest mean coefficients, is heavily enriched with features coming from the molecular layer, while the *top-20 *is basically comprised of image features. The HPCAL1.1 gene was encountered in all of the unified signatures with the exception of b.i, where CDC37L1 was observed.

Regarding the imaging features, they can contribute to the construction of reliable operators, that consolidate the wealth of morphological information, critical for the task of the dynamic description of the undergoing transformations of biological procedures in relation to disease manifestation. Moreover they may represent reliable macroscopic candidates for assessment of disease molecular subtypes, something that could be strengthened if covariance-based association of those markers with the molecular markers is undertaken. However in order that this analysis is biologically insightful and of practical use, large populations of cohorts are needed to check the consistency of the findings.

The molecular part of the feature set comprises genes which imply promotion of tumorigenesis, angiogensis, and protein endoplasmic misfolding, that induces stress response repair signals, xenobiotic metabolism, and so forth, namely, involvement of cellular modular functions. These functions are known to be associated with the carcinogenic aberrant course in particular for aggressive cancers like CM. Indicatively, FOX1 possesses an established role in myogenic growth, differentiation, and blood angiogenesis. Defective function of FOX1 is incriminated for rhabdomyosarcoma type 2, a highly malignant tumour of striated muscle derived from primitive mesenchymal cells, which is a cancer model that is evolutionary close to skin cells [36]. CDC37L1 is a cochaperone that binds to numerous proteins and promotes their interaction with Hsp70 and Hsp90, whose aberrant function suggests an endoplasmic reticulum stress response as a result of protein misfolding stress [37]. MMP1 is implicated in the breakdown of extracellular matrix in normal, physiological processes, such as embryonic development, reproduction, tissue remodeling, and blood coagulation, as well as in disease processes such as arthritis and the metastatic procedure [38], for which melanoma cells present a high potency. Interestingly, HPCAL1 encodes an extracellular protein, which is a member of neuron-specific, calcium-binding, protein family, found in the retina and brain [39]. This finding is consistent with the fact that skin and neural cells, especially the cancer ones that are dedifferentiated, have common progenitor lines.

### 5.5. Future Work

In this study, the direct comparison between RF and the linear information-oriented methods, regarding the stability of the proposed predictor set for biomarker discovery, was based on an empirical cut-off, namely, the top-20 features, as surmised by the LDA method. Subsequently, mode membership (image or microarrays) in the 20 feature set was examined for the two schemes: *top-means* and top-20. Still, this approach could be further developed to implement automated feature selection, exploiting statistically derived decision cut-offs. It can also be extended to enable juxtaposition with the automated filtering that occurs in the feature selection with RF, which is depicted at the 3rd column of Tables 1 and 2. In addition, the signatures derived by the unified datasets could be compared with clinical multimodal data stemming from the same set of patients. In this way, the impact of the imputation methods in the creation of the synthetic dataset could be cross-evaluated, with the performance of other data analysis methods, such as canonical correlation analysis or variations of such methods.

## Supplementary Material


**Paragraph description for Supplementary 1: **PCA representations for the 6 different datasets, classifying melanomas from healthy donors, namely 2 datasets corresponding toeither the microarray(o.m) or the dermoscopic data(o.i), plus 4 integrated datasets, applying the missing value imputation schemes, defined in page 5.
**Paragraph description for Supplementary 2: **LDA representations for the 6 different datasets, classifying melanomas from healthy donors, namely 2 datasets corresponding to either microarray (o.m) or dermoscopic data (o.i), plus 4 integrated datasets, applying the missing value imputation schemes, defined in page 5.

## Figures and Tables

**Table 1:** Top features (genes) selected after 50 repetitions of the 10-fold cross-validation modeling for the best-filtered case in each of the tree datasets (o.m, m.i, and nr.i along with the frequencies of appearance).

Feature	Freq.	Feature	Freq.	Feature	Freq.
(o.m)	(o.m)	(m.i)	(m.i)	(nr.i)	(nr.i)
CDC37L1	47	NEIL1	4	CDC37L1	49
RRAS2	34	IFI16	3	RRAS2	2
SLC7A8	18	CTDSPL	2		
HPCAL1	14	DLK2	2		
IFT81	8	NADK	2		
SSBP2	6	OR2A9P	2		
GIPC2	5	PIK3C2G	2		
CTDSPL	3				

**Table 2:** Top features selected at the tolerance-filtered case.

Feature	Freq.	Feature	Freq.	Feature	Freq.
(o.m)	(o.m)	(m.i)	(m.i)	(nr.i)	(nr.i)
CDC37L1	45	PARD3	5	CDC37L1	40
RRAS2	25	ACOT9	3	RRAS2	6
SLC7A8	17	CYP4F3	3	HPCAL1.	2
HPCAL1.1	10	FZD10	3	SSBP2	2
IFT81	6	NEIL1	3		
GIPC2	5	ACADL	2		
CTDSPL	4	MTUS1	2		
NEIL1	4	PER3	2		
SSBP2	3	PPP2R3A	2		
SMAD5OS	2	SMAD5OS	2		

**Table 3:** Top-20 LDA features after 100 repetitions.

o.m	o.i	m.i *top-means *	nr.i *top-means *	nr.i top-20	nr.i top-20 freq.	u.i *top-means *	u.i top-20	u.i top-20 freq.	b.i *top-means *	b.i top-20	b.i top-20 freq.
ABCD1	I.mean	I.mean	HPCAL1.1	L.mean	78	HPCAL1.1	HPCAL1.1	75	CDC37L1	L.std	77
CRIM1.1	mean.R	mean.R	MMP1	mean.G	75	Grad.std	mean.B	69	MMP1	I.std	74
UPP1	mean.G	mean.G	CDC37L1	I.mean	71	CDC37L1	I.mean	63	HPCAL1.1	I.mean	70
GSTT1	mean.B	mean.B	C2orf68	I.std	70	Grad.mean	mean.G	61	ZSCAN18	mean.G	70
HPCAL1.1	A.mean	A.mean	A.mean	L.std	68	MMP1	Grad.std	59	C2orf68	L.mean	69
ZSCAN18	L.std	L.std	ID4	mean.B	63	ID4	L.std	59	ALOX12	A.mean	68
IFI16	I.std	I.std	ZSCAN18	std.B	61	SSBP2	std.B	58	PARD3	mean.B	67
SHARPIN	S.mean	S.mean	MTUS1	std.G	61	NRP2	I.std	57	ID4	mean.R	66
TMEM80	std.R	std.R	SSBP2	mean.R	60	IFT81	L.mean	56	MTUS1	S.mean	61
HOMER3.1	S.std	S.std	ALOX12	A.mean	59	FOXO1	CDC37L1	55	IFT81	std.B	61
PLAUR.1	A.std	A.std	NCAPH	GMSM.mean	54	ZSCAN18	A.mean	53	NCAPH	std.G	59
SNTB1	std.B	std.B	ANG	PERIMETER	54	RRAS2	B.std	53	NRP2	std.R	59
SFRP1.1	std.G	std.G	std.B	A.std	53	GPRIN2	std.G	52	CYP3A5.1	PERIMETER	58
MTUS1	B.mean	ABCD1	NRP2	HPCAL1.1	53	MTUS1	PERIMETER	51	SSBP2	DISSIMILARITY	54
RPRM	L.mean	B.mean	PARD3	S.mean	52	ALOX12	Grad.mean	49	ANG	A.std	50
FZD7	H.std	UPP1	CYP3A5.1	B.mean	50	mean.B	std.R	48	FOXO1	CDC37L1	50
SRC	H.mean	L.mean	ABCD1	std.R	50	NCAPH	A.std	45	C12orf29	GMSM.mean	49
MMP1	Distance.std	HPCAL1.1	BAZ1B	COMPLEXITY	48	NEIL1	mean.R	45	BAZ1B	B.std	47
LAMB4	PERIMETER	ZSCAN18	C12orf29	S.std	48	IFI16	DISSIMILARITY	44	ABCD1	B.mean	43
NEK2	COMPLEXITY	IFI16	IFT81	B.std	45	SLC7A8	S.mean	44	MAOA.1	COMPLEXITY	43
