# Supplementary material for: Integration of High-Volume Molecular and Imaging Data for Composite Biomarker Discovery in the Study of Melanoma
Source: Biomed Res Int. 2014 Jan 16;2014:145243. doi: 10.1155/2014/145243 (PMC3914284; doi:10.1155/2014/145243)
Supplement: Supplementary file 1 — Paragraph description for Supplementary 1: PCA representations for the 6 different datasets, classifying melanomas from healthy donors, namely 2 datasets corresponding toeither the microarray(o.m) or the dermoscopic data(o.i), plus 4 integrated datasets, applying the missing value imputation schemes, defined in page 5. Paragraph description for Supplementary 2: LDA representations for the 6 different datasets, classifying melanomas from healthy donors, namely 2 datasets corresponding to either microarray (o.m) or dermoscopic data (o.i), plus 4 integrated datasets, applying the missing value imputation schemes, defined in page 5. [file 145243.f1.pdf]

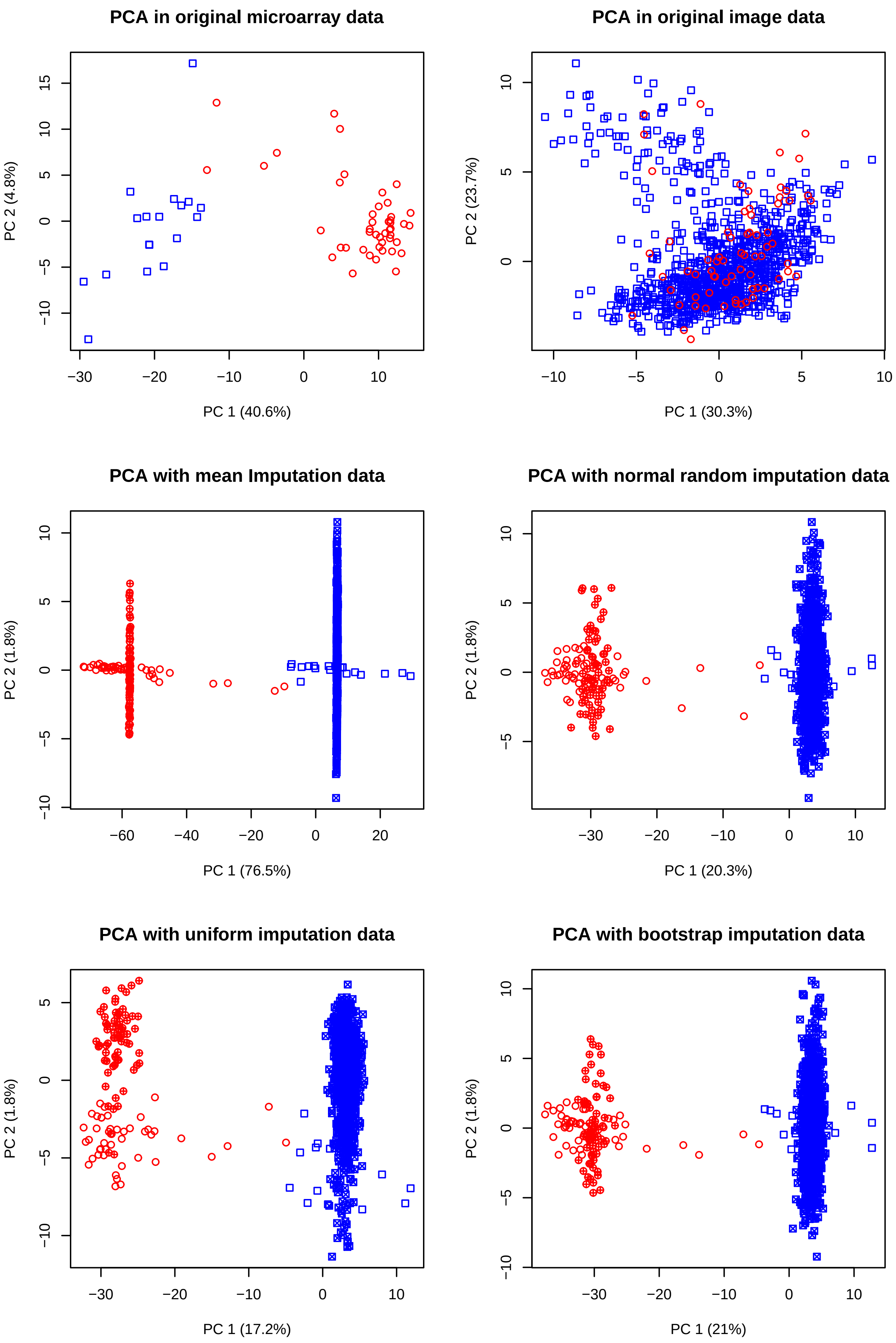
PCA in original microarray data
PC 1 (40.6%)
PC 2 (4.8%)
PCA in original image data
PC 1 (30.3%)
PC 2 (23.7%)
PCA with mean Imputation data
PC 1 (76.5%)
PC 2 (1.8%)
PCA with normal random imputation data
PC 1 (20.3%)
PC 2 (1.8%)
PCA with uniform imputation data
PC 1 (17.2%)
PC 2 (1.8%)
PCA with bootstrap imputation data
PC 1 (21%)
PC 2 (1.8%)
