# Supplementary material for: Integration of High-Volume Molecular and Imaging Data for Composite Biomarker Discovery in the Study of Melanoma
Source: Biomed Res Int. 2014 Jan 16;2014:145243. doi: 10.1155/2014/145243 (PMC3914284; doi:10.1155/2014/145243)
Supplement: Supplementary file 2 [file 145243.f2.pdf]

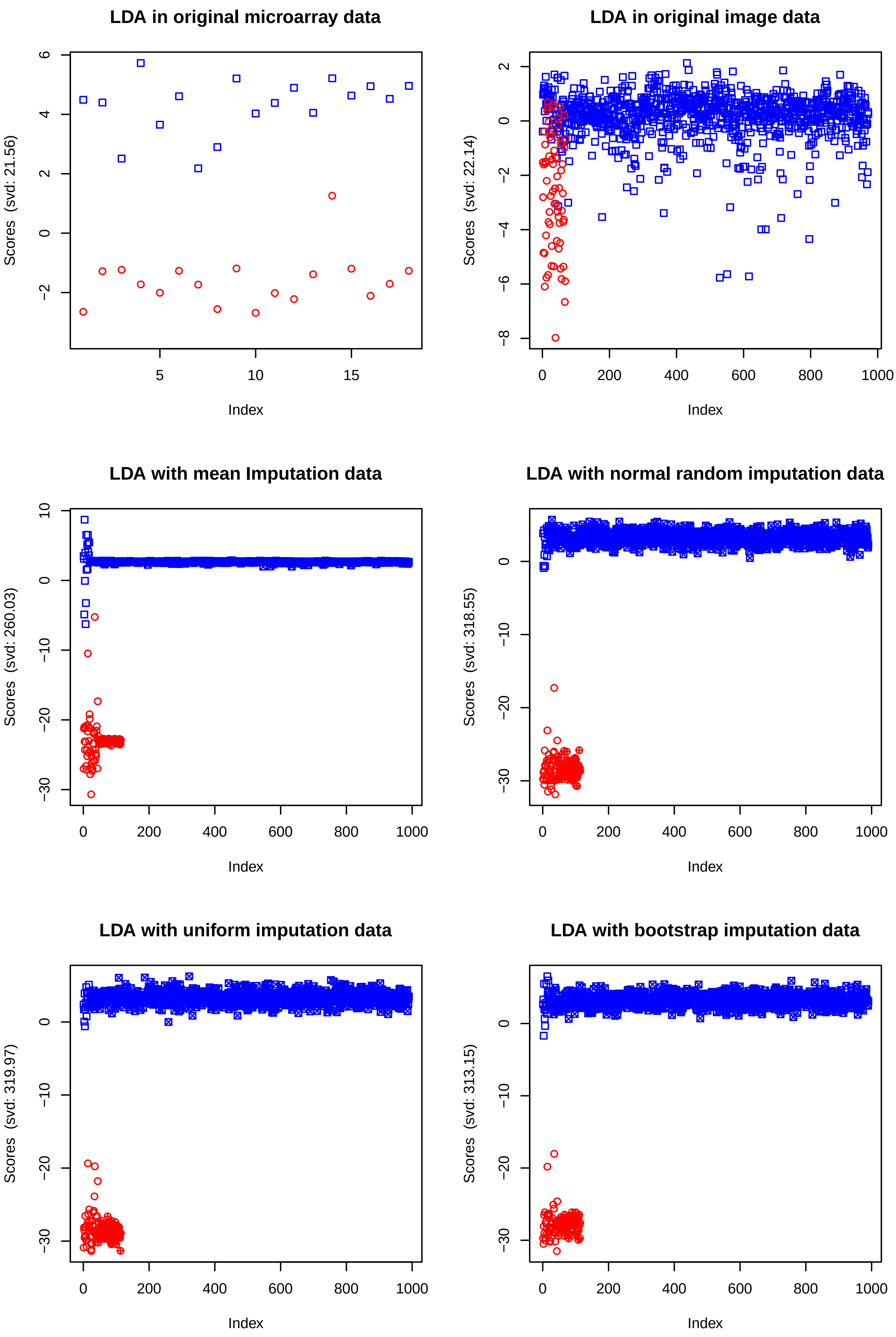
LDA in original microarray data
Scores (svd: 21.56)
Index
LDA in original image data
Scores (svd: 22.14)
Index
LDA with mean Imputation data
Scores (svd: 260.03)
Index
LDA with normal random imputation data
Scores (svd: 318.55)
Index
LDA with uniform imputation data
Scores (svd: 319.97)
Index
LDA with bootstrap imputation data
Scores (svd: 313.15)
Index
